# Bidirectional modulation of synaptic transmission by insulin-like growth factor-I

**DOI:** 10.3389/fncel.2024.1390663

**Published:** 2024-06-07

**Authors:** José Antonio Noriega-Prieto, Laura Eva Maglio, Paloma Perez-Domper, José Carlos Dávila, Antonia Gutiérrez, Ignacio Torres-Alemán, David Fernández de Sevilla

**Affiliations:** ^1^Departamento de Anatomía, Histología y Neurociencia, Facultad de Medicina, Universidad Autónoma de Madrid, Madrid, Spain; ^2^Department of Neuroscience, University of Minnesota, Minneapolis, MN, United States; ^3^Centro de Investigaciones Biomédicas en Red Enfermedades Neurodegenerativas (CIBERNED), Madrid, Spain; ^4^Instituto Cajal (CSIC), Madrid, Spain; ^5^Departamento Biología Celular, Genética y Fisiología. Facultad de Ciencias, Instituto de Investigación Biomédica de Málaga, Universidad de Málaga, Málaga, Spain; ^6^Achucarro Basque Center for Neuroscience, Leioa, Spain; ^7^Ikerbasque Science Foundation, Bilbao, Spain

**Keywords:** insulin-like growth factor-I, LTP, LTD, spike-timing dependent plasticity, Hebbian plasticity

## Abstract

Insulin-like growth factor-I (IGF-I) plays a key role in the modulation of synaptic plasticity and is an essential factor in learning and memory processes. However, during aging, IGF-I levels are decreased, and the effect of this decrease in the induction of synaptic plasticity remains unknown. Here we show that the induction of N-methyl-D-aspartate receptor (NMDAR)-dependent long-term potentiation (LTP) at layer 2/3 pyramidal neurons (PNs) of the mouse barrel cortex is favored or prevented by IGF-I (10 nM) or IGF-I (7 nM), respectively, when IGF-I is applied 1 h before the induction of Hebbian LTP. Analyzing the cellular basis of this bidirectional control of synaptic plasticity, we observed that while 10 nM IGF-I generates LTP (LTP_IGF-I_) of the post-synaptic potentials (PSPs) by inducing long-term depression (LTD) of the inhibitory post-synaptic currents (IPSCs), 7 nM IGF-I generates LTD of the PSPs (LTD_IGF-I_) by inducing LTD of the excitatory post-synaptic currents (EPSCs). This bidirectional effect of IGF-I is supported by the observation of IGF-IR immunoreactivity at both excitatory and inhibitory synapses. Therefore, IGF-I controls the induction of Hebbian NMDAR-dependent plasticity depending on its concentration, revealing novel cellular mechanisms of IGF-I on synaptic plasticity and in the learning and memory machinery of the brain.

## Introduction

Insulin-like growth factor-1 (IGF-I) is a peptide with well-known trophic functions in the brain, but its neuromodulatory role is still under study. It is well documented that IGF-I regulates neuronal firing (Carro et al., 2000; Nuñez et al., 2003; Gazit et al., 2016), and modulates synaptic transmission in several areas of the central nervous system (CNS), such as hippocampus or cerebellum (Nilsson et al., 1988; Araujo et al., 1989; Castro-Alamancos and Torres-Aleman, 1993; Seto et al., 2002; Fernando Maya-Vetencourt et al., 2012). IGF-I is also able to enhance glutamatergic synaptic transmission in rat hippocampal slices of juvenile animals through a mechanism that involves α-amino-3-hydroxy-5-methyl-4-isoxazolepropionic acid (AMPA) but not N-methyl-D-aspartate (NMDA) postsynaptic receptors, probably through phosphatidylinositol-3-kinase (PI3K) activation (Ramsey et al., 2005). However, in young adult and old rats, IGF-I significantly increases both AMPA and NMDA-mediated synaptic transmission by a postsynaptic mechanism (Molina et al., 2013). Additionally, the increase in expression levels of NMDA receptor subunits 2A and 2B at the hippocampus by IGF-I in aged rats (Sonntag et al., 2000) may facilitate the induction of long-term potentiation (LTP). Moreover, a reduction of inhibitory synaptic transmission dependent on the activation of the IGF-I receptor (IGF-IR) on astrocytes (Noriega-Prieto et al., 2021), and on the synthesis of nitric oxide (Noriega-Prieto et al., 2022) has been demonstrated in the barrel cortex.

IGF-I is actively transported to the CNS from plasma through the blood-brain-barrier (Nishijima et al., 2010), and is also locally produced by neurons and glial cells (Quesada et al., 2007; Rodriguez-Perez et al., 2016). Therefore, IGF-I levels in specific brain areas depend both on its local release and on its uptake from the circulation. Accordingly, the entry of serum IGF-I into specific brain areas is more significant during periods of increased neuronal activity or physical activity (Carro et al., 2000; Nishijima et al., 2010). Therefore, different brain areas may have different IGF-I levels depending on their levels of neuronal activity. Moreover, aging is associated with lower serum IGF-I levels (Breese et al., 1991) and impaired brain IGF-I activity (Muller et al., 2012), suggesting that IGF-I levels within as yet an undetermined range are essential for proper brain functioning. Thus, while brain IGF-I function varies according to age and brain activity, it is not known whether differing IGF-I concentrations will have distinct effects on the modulation of synaptic transmission, a key aspect of brain function.

In the present work, we have investigated the effect of different IGF-I levels in the modulation of the threshold of NMDAR-dependent LTP at layer 2/3 pyramidal neurons (L2/3 PNs) of the mouse barrel cortex. We demonstrate that 1 h after application of 10 nM IGF-I, Hebbian LTP is favored by reducing the number of pairings required to induce it, as well as the magnitude of the potentiation, whereas its induction is prevented after 7 nM IGF-I. Analyzing the underlying effects, we found that 10 nM IGF-I generates LTP of the PSPs (LTP_IGF-I_) by inducing long-term depression (LTD) of inhibitory synaptic transmission and STP of the EPSCs. Conversely, 7 nM IGF-I generates LTD of the PSPs (LTD_IGF-I_) by inducing LTD of the EPSCs. Therefore, our results demonstrate that IGF-I levels are essential for the induction of Hebbian long-term synaptic plasticity at the barrel cortex by inducing a bidirectional long-term modulation of synaptic transmission, which may have important consequences in learning and memory processes in the mouse.

## Materials and methods

All animal procedures were approved by the Ethical Committee of the Universidad Autónoma of Madrid, and Cajal Institute and are in accordance with Spanish (R.D. 53/2013) and European Community Directives (2010/63/EU), which promote the animal welfare. Male C57BL/6J mice were housed in standard laboratory cages with *ad libitum* access to water and food in temperature- and humidity-controlled rooms under a 12 h/12 h light/dark cycle with up to five animals per cage and were used for slice electrophysiology. Mice from different litters were used to increase the reproducibility of the experiments.

### Electrophysiology in brains slices

C57BL6/J mice (12–18 days old) were slightly anesthetized with isoflurane, decapitated, and the brain quickly removed and immersed in ice-cold high-sucrose “cutting solution” containing (in mM): 189.0 sucrose, 10.0 glucose, 26.0 NaHCO_3_, 3.0 KCl, 5.0 Mg_2_SO_4_, 0.1 CaCl_2_, 1.25 NaH_2_PO_4_·2H_2_O. Coronal slices (350 μm thick) were cut with a Vibratome (Leica VT 1200S), then slices were incubated (>1 h, at room temperature, 25–27°C) in artificial cerebrospinal fluid (ACSF) which containing (in mM): 124.00 NaCl, 2.69 KCl, 1.25 KH_2_PO_4_, 2.00 Mg_2_SO_4_, 26.00 NaHCO_3_, 2.00 CaCl_2_, 10.00 glucose, 0.4 ascorbic acid and 0.3 sodium pyruvate. The pH was stabilized at 7.4 by bubbling the ACSF with carbogen (95% O_2_, 5% CO_2_). Slices were transferred to a 2 mL chamber fixed to an upright microscope stage (BX51WI; Olympus, Tokyo, Japan) equipped with infrared differential interference contrast video (DIC) microscopy and a 40X water-immersion objective and superfused at room temperature with carbogen-bubbled ACSF (2 mL/min). Cells were visualized under an Olympus BX50WI microscope. Patch-clamp recordings from the soma of L2/3 PNs of the barrel cortex were performed in the whole-cell voltage-clamp and current-clamp configurations. Patch pipettes were made from 1.50 OD/0.86 ID borosilicate glass capillaries (GC150F/10; Harvard Apparatus) using a P97 micropipette puller (Sutter Instruments) and had resistances of 4–8 MΩ when filled with an internal solution that contained (in mM): 120 K-Gluconate, 10 KCl, 10 HEPES, 0.5 EGTA, 4 Na_2_-ATP, and 0.3 Na_3_-GTP, 10 NaCl buffered to pH 7.2–7.3 with KOH. (280 mOsm) Recordings were performed in the current- or voltage-clamp modes using a Cornerstone PC-ONE amplifier (DAGAN, Minneapolis, MN). Pipettes were placed with a mechanical micromanipulator (Narishige, Tokyo, Japan). The holding potential was adjusted to −60 mV, and the series resistance was compensated to ~70%. L2/3 PNs located over the barrels (layer 4) were accepted only when the seal resistance was >1 GΩ and the series resistance (10–20 MΩ) did not change (>10%) during the experiment. Data were low-pass filtered at 3.0 kHz and sampled at 10.0 kHz, through an Axon Digidata 1440A interface board (Molecular Devices, Sunnyvale, CA). The pClamp software (Molecular Devices) was used to acquire the data. Chemicals were purchased from Sigma-Aldrich and Tocris Bioscience.

Synaptic responses were evoked with Pt/Ir concentric bipolar (OP 200 μm, IP 50 μm, FHC) stimulating electrodes (0.1 ms and 20–100 μA) connected by 2 silver-chloride wires to a Grass S88 stimulator and stimulus isolation unit (Quincy, United States). The stimulating electrode was placed at layer 4 of the barrel cortex. Single pulses (100 μs duration and 20–100 μA) were continuously delivered at 0.33 Hz.

### Spike-timing dependent plasticity, Hebbian LTP

**Figure 1:**
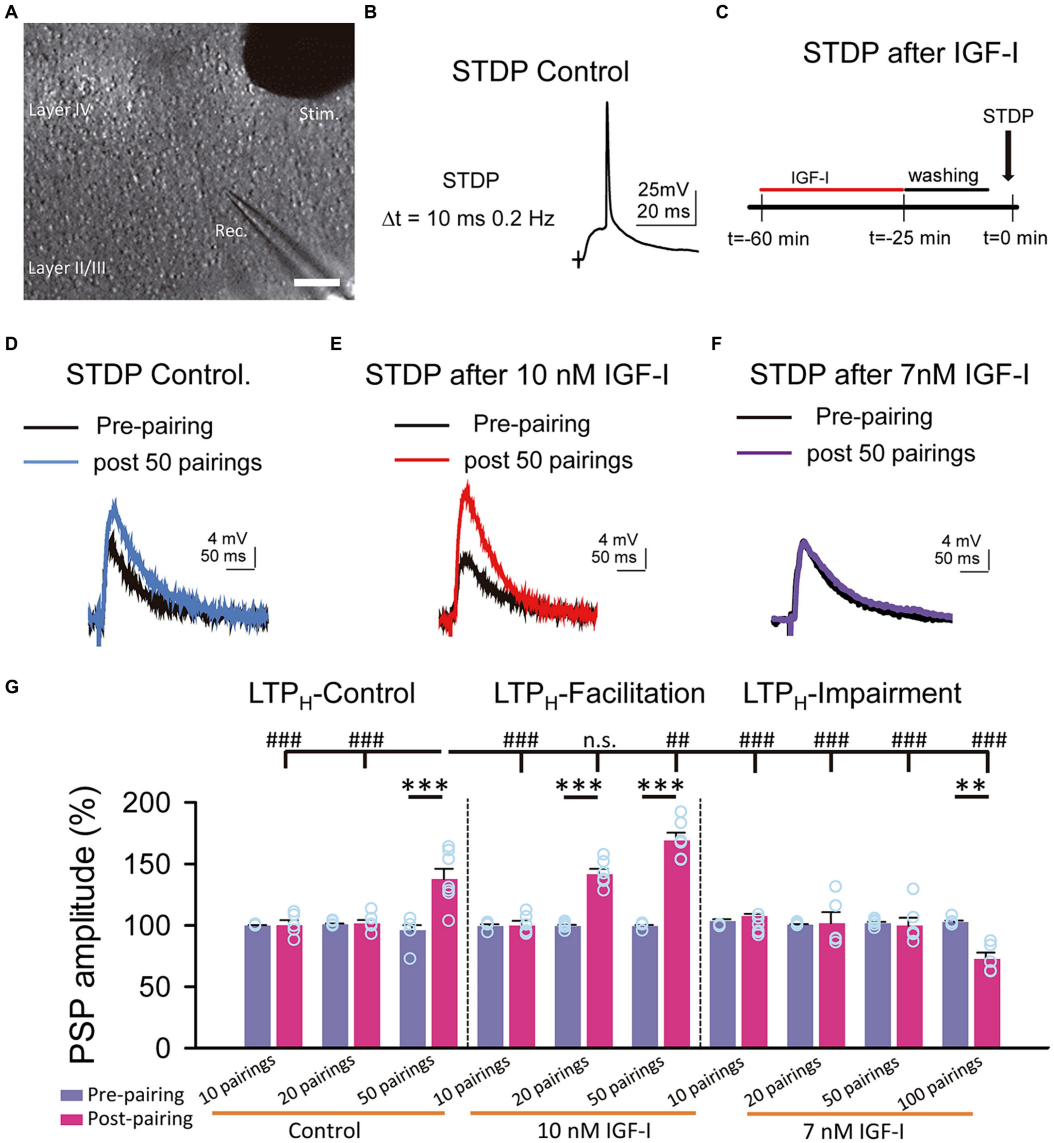
Hebbian LTP is favored and impaired by IGF-I 10 nM and 7 nM, respectively. **(A)** DIC image showing the recording (Rec.) located in layer II/III and the stimulation (Stim.) electrodes locate in layer IV in a slice (scale bar 100 μm). **(B)** Representative responses recorded (PSP followed by an AP with a 10 ms delay and a frequency of 0.2 Hz) during the STDP protocol in control. **(C)** Time course scheme showing the IGF-I exposure, washout and STDP induction (black arrow). **(D)** Superimposed representative PSPs (pre-pairing, black trace) and 40 min after 50 pairings (post 50 pairings, blue trace) in the control experiment of STDP. **(E)** Same as **D** but when 10 nM IGF-I was bath applied before the STPD. **(F)** Same as **D** but when 7 nM IGF-I was bath applied before the STPD. **(G)** Bar plot showing the PSP peak amplitude as percentage of the control before (pre-paring, blue bars) and 40 min after (post-pairing, red bars) the application of the STDP protocol in which 10, 20 and 50 parings were applied in control (LTP_H_-Control), or after 10 nM IGF-I (LTP_H_-facilitation) or after 10, 20, 50 and 100 parings 7 nM IGF-I (LTP_H_-impairment). ^*^p < 0.05, ^**^p < 0.01, and ^***^p < 0.001; student’s paired *t*-test. ^#^*p* < 0.01, ^##^*p* < 0.01, and ^###^*p* < 0.001; one-way ANOVA with *post hoc* Holm–Sidak; n.s., nonsignificant (*p* > 0.05).

In some experiments, after applying IGF-I as described above, spike-timing dependent plasticity (STDP) was induced (Figure 1). Induction of STDP was achieved by pairing pre- and postsynaptic action potentials 10 ms away. Presynaptic action potentials were evoked by electrical stimulation of basal afferent inputs. Stimulus intensity was adjusted to obtain 3–5 mV PSPs responses. Postsynaptic action potentials were elicited by a brief current injection through the recording pipette (5 ms, 200–400 pA). Basal PSPs were recorded for 10 min before the pre-post associations were induced (30, 20, 50 and 100 pairings were studied). After that, PSPs were recorded for another 50–60 min. Amplitude of PSPs 5 min before (−5 to 0 min interval) and 50 min after (45 to 50 min interval) the LTP_H_ induction protocol were measured to compare the extent of the PSP potentiation.

### IGF-I effects on synaptic transmission recordings

**Figure 2:**
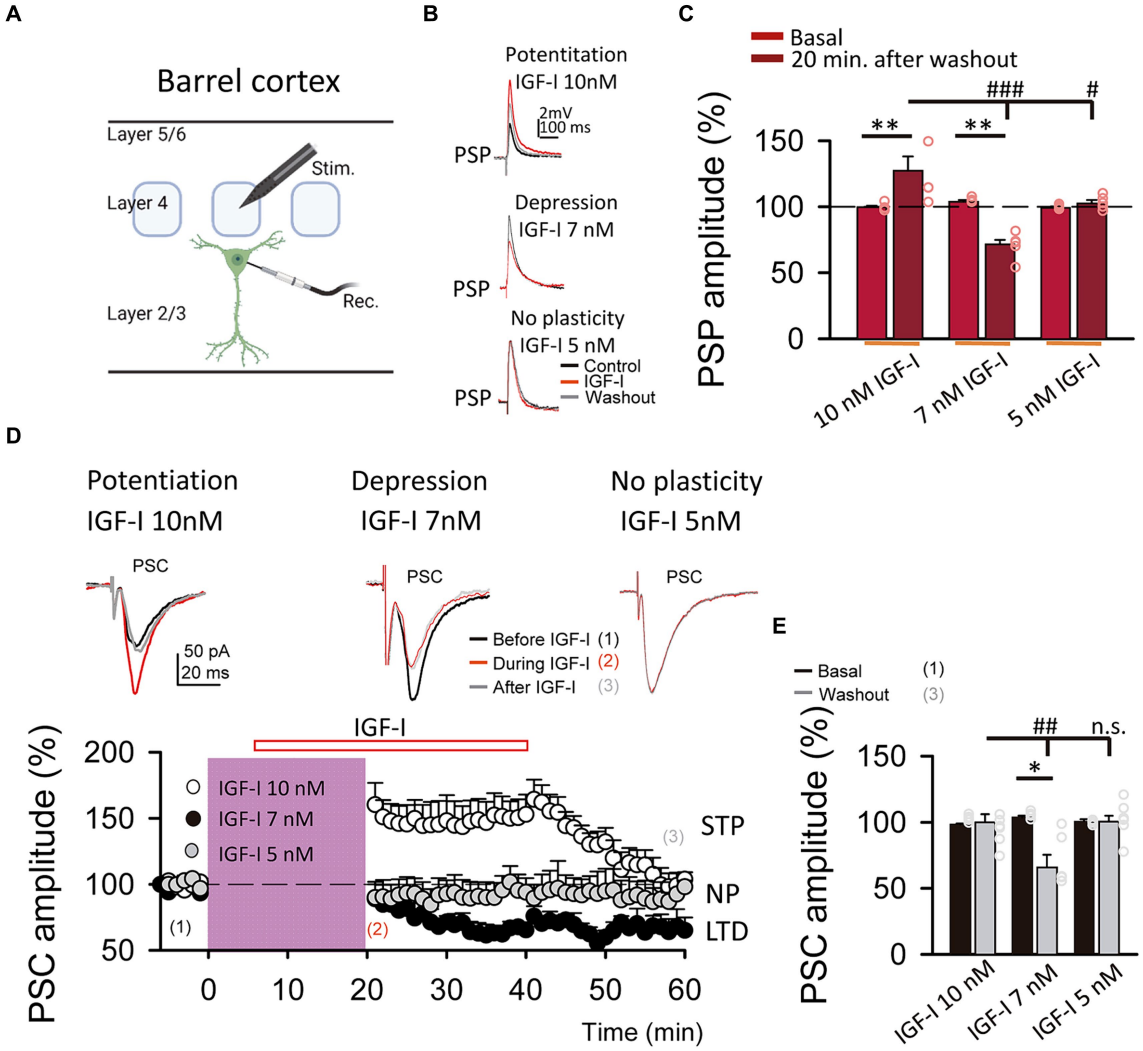
LTP and LTD of the postsynaptic potentials are induced by IGF-I 10 nM and 7 nM, respectively. **(A)** Schematic representation of layer 2/3 pyramidal neuron recording (Rec.) and the stimulation electrode (Stim.) in the barrel cortex (scheme was performed with BioRender). **(B top)** Superimposed representative PSPs recorded before (black trace, control), during (red trace, IGF-I) and 40 min after 10 nM IGF-I washout (grey trace, washout), showing the induction of PSP potentiation by IGF-I (LTP_IGF1_). **(B middle)** Superimposed representative PSPs recorded before (black trace, control), during (red trace, IGF-I) and 40 min after 7 nM IGF-I washout (grey trace, washout), showing the induction of PSP potentiation by IGF-I (LTP_IGF1_). **(B bottom)** Superimposed representative PSPs recorded before (black trace, control), during (red trace, IGF-I) and 40 min after 5 nM IGF-I washout (grey trace, washout), showing the induction of PSP depression by IGF-I (LTD_IGF1_). **(C)** Bar plot showing the PSP peak amplitude as percentage of the control before (control) and 40 min after IGF-I (washout) 10 nM, 7 nM and 5 nM IGF-I. **(D top)** Superimposed representative PSCs recorded before (black trace, control), during (red trace, during IGF-I) and 40 min after IGF-I washout (grey trace, after IGF-I), showing the induction of PSP potentiation by 10 nM IGF-I, the PSP depression by IGF-I 7 nM and the no plasticity by IGF-I 5 nM. **(D bottom)** Time course of the PSC peak amplitude expressed as percentage of control before, during and after washing out the IGF-I 10 nM (white circles, short term potentiation STP) 7 nM (black circles, LTD) and 5 nM (grey circles, no plasticity, NP). **(E)** Bargraph showing the PSC change percentage showed in **D bottom**. ^*^p < 0.05, ^**^p < 0.01, and ^***^p < 0.001; student’s paired *t*-test. ^#^*p* < 0.01, ^##^*p* < 0.01, and ^###^*p* < 0.001; one-way ANOVA with *post hoc* Holm–Sidak; n.s., nonsignificant (*p* > 0.05).

**Figure 3:**
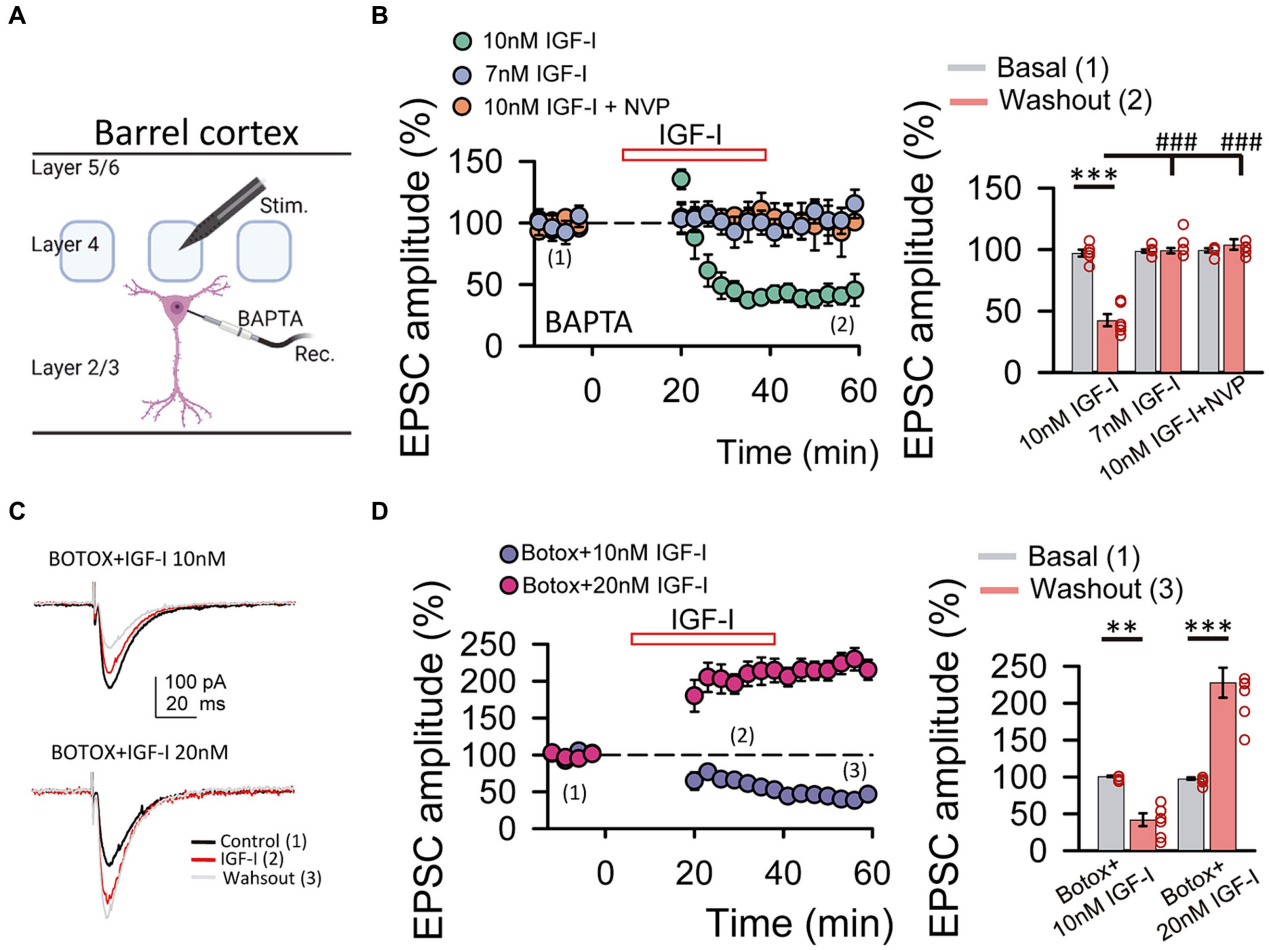
IGF-I 10 nM induces LTD when postsynaptic calcium increases and endosome exocytosis are prevented by BAPTA and BOTOX, respectively. **(A)** Scheme showing the recording of layer 2/3 pyramidal neuron with a patch pipette containing 20 mM BAPTA and the stimulating electrode located at layer 4 of the barrel cortex (scheme was performed with BioRender). **(B left)** Time course of the EPSCs peak amplitude as percentage of the control under BAPTA in the patch pipette before, during (IGF-I, red empty bar) and after washout of 10 nM IGF-I (green circles), 7 nM IGF-I (blue circles) and in the presence of 10 nM IGF-I + NVP (orange circles). **(B right)** Bar plot showing the EPSC peak amplitude as percentage of the control under BAPTA in the patch pipette before (basal, grey bars) and after (light red bars) washout of 10 nM IGF-I, 7 nM IGF-I and 10 nM IGF-I + NVP. Note that under BAPTA LTD is induced by 10 nM IGF-I. **(C left top)** Superimposed representative EPSCs recorded before (control, black trace), during (IGF-I, red trace) and 40 min after washout of 10 nM IGF-I (washout, grey trace) with a BOTOX 1 μM containing intracellular solution in the patch pipette (Botox + IGF-I 10 nM). **(C left bottom)** Same as top but increasing IGF-I concentration to 20 mM. **(C right)** Bar plot showing the EPSC peak amplitude as percentage of the control under Botox in the patch pipette before (control, dark blue bars), during (IGF-I, middle blue bars) and 40 min after washout of IGF-I (washout, green bars) in normal ACSF (BAPTA bars) when applying IGF-I 10 nM (left bars, Botox + 10 nM IGF-I) and 20 nM (right bars, Botox + 20 nM IGF-I). Note that under Botox, LTD is induced by 10 nM IGF-I and that LTP can be restored by increasing the IGF-I concentration to 20 nM. ^*^p < 0.05, ^**^p < 0.01, and ^***^p < 0.001; student’s paired *t*-test. ^#^*p* < 0.01, ^##^*p* < 0.01, and ^###^*p* < 0.001; one-way ANOVA with *post hoc* Holm–Sidak; n.s., nonsignificant (*p* > 0.05).

In all experiments, basal postsynaptic currents (PSCs) were recorded for 5 min at 0.33 Hz (voltage-clamp configuration), then basal postsynaptic potentials (PSPs) were recorded at 0.2 Hz (current-clamp configuration). After, the stimulation intensity was increased until ≈10% of the responses recorded were suprathreshold. Following IGF-I (Preprotech) was added to the bath and the PSPs were recording for 15 min. Then, we switched back to voltage-clamp, returning the intensity and frequency to that used for the baseline recording, and we recorded the PSCs for 20 min. After that, we washed the IGF-I and recorded PSCs during 20 min after washout IGF-I. We switched each 10 min from voltage-clamp to current-clamp to check the amplitude of the PSPs (during 2 min) after IGF-I washout. We added different IGF-I concentration (5, 7 and 10 nM) Plots of the changes induced by IGF-I on EPSCs peak amplitude (percentage from control) versus time were constructed (Figure 2). The same experiment was performed in present to the IGF-I antagonist receptor 7-[cis-3-(1-178azetidinylmethyl)cyclobutyl]-5-[(3-phenylmethoxy)phenyl]-7H-pyrrolo[2,3-]pyrimidin-179 4-amine (NVP-AEW 541, 40 μM; Cayman Chemicals) plus IGF-I. In some experiments, the intracellular solutions could also either contain: 1,2-Bis(2-aminophenoxy) ethane-N,N,N′,N′-tetra acetic acid (BAPTA; 20 mM) or light chain of the B-type botulinum toxin (BOTOX, 1 μM) (Figure 3).

### Synaptic stimulation increased

**Figure 4:**
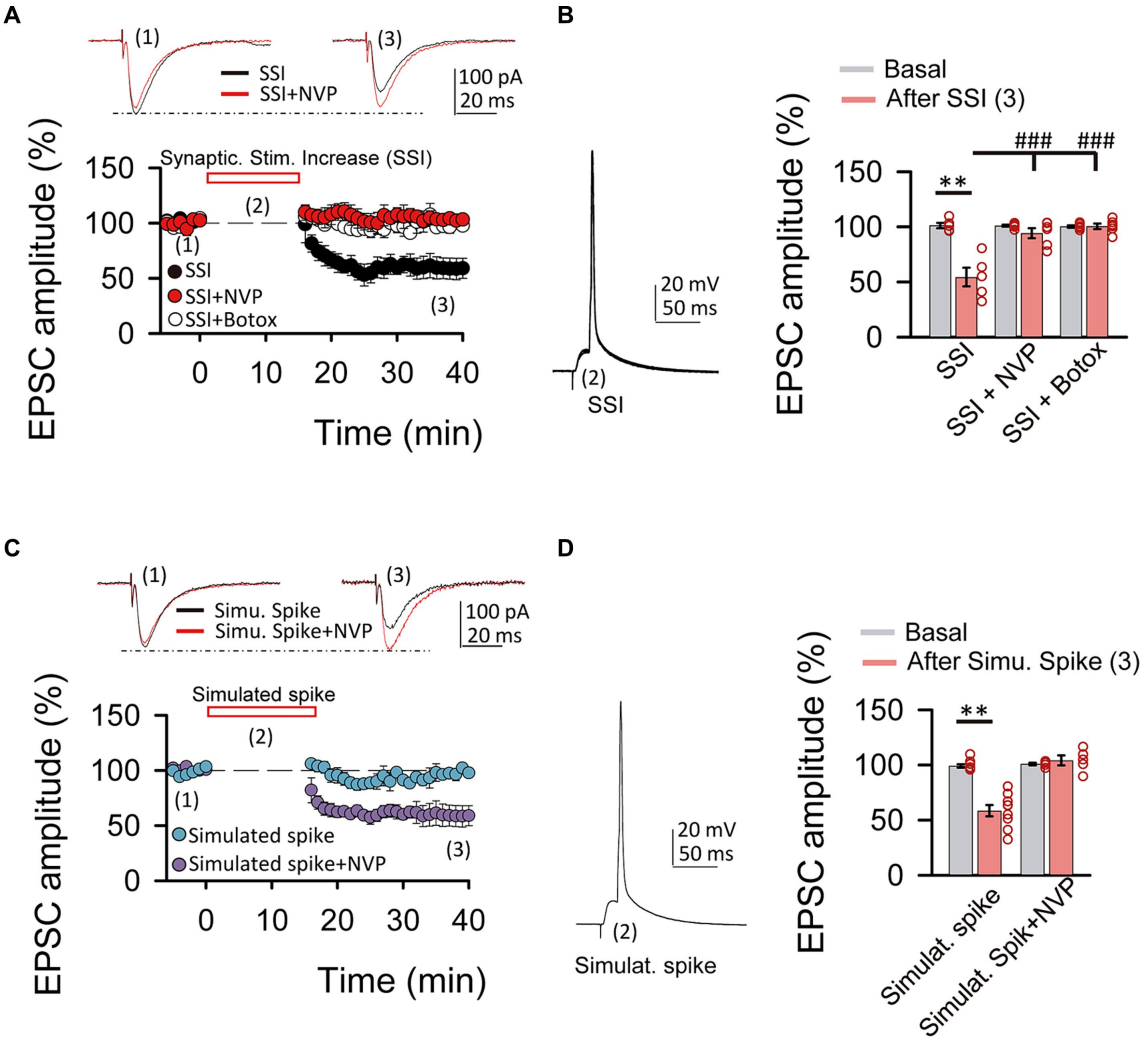
Spiking activity is able to induce LTD_IGF1_ without exogenous application of IGF-I. **(A top)** Superimposed representative EPSCs recorded before (1) and 40 min after (3) spiking activity evoked by synaptic stimulation increase (2, SSI) in normal ACSF (black trace, SSI) and under NVP (red trace, SSI + NVP). **(A bottom)** Time course of the EPSC recorded before, during and after SSI in control (SSI, black circles), under NVP (SSI + NVP, white circles) and under Botox (SSI + botox, red circles). **(B left)** Representative spiking activity evoked by the synaptic stimulation increase (2). **(B right)** Bar plot showing the EPSC peak amplitude as percentage of the control after SSI in ASCF (SSI, black bar), under NVP (SSI + NVP, white bar) and under Botox (SSI + Botox, red bar). Note that SSI is able to induce an LTD that is prevented by both NVP and Botox and then is very similar to LTD_IGF1_. **(C top)** Superimposed representative EPSCs recorded before (1) and 40 min after (3) spiking activity evoked by simulated spike (2, simulated spike) in normal ACSF (black trace, stimu. spike) and under NVP (red trace, stimu. spike + NVP). **(C bottom)** Time course of the EPSC recorded before, during and after SSI in control (SSI, black circles), under NVP (SSI + NVP, white circles). **(D left)** Representative simulated spike (2). **(D right)** Bar plot showing the EPSC peak amplitude as percentage of the control after simulates spike in ASCF (stimulat. Spike, purple bar), under NVP (stimulat. spike + NVP, blue bar). Note that simulated spikes are able to induce an LTD that is prevented by NVP similar to LTD_IGF1._ **(E)** Plot of the variance (1/CV^2^, where CV is coefficient of variation) as a function of the mean peak EPSC amplitude during simulated spike protocol normalized to control conditions (Mr). Note that the values were grouped following the diagonal suggesting that LTD was due to a change in presynaptic release properties. ^*^p < 0.05, ^**^p < 0.01, and ^***^p < 0.001; student’s paired *t*-test. ^#^*p* < 0.01, ^##^*p* < 0.01, and ^###^*p* < 0.001; one-way ANOVA with *post hoc* Holm–Sidak; n.s., nonsignificant (*p* > 0.05).

Synaptic stimulation increased (SSI), after 5 min of stable baseline of EPSCs in voltage-clamp, the recording was switched to the current-clamp and the stimulation intensity was increased to values in which an EPSP followed by an AP was triggered during 15 min at 0.2 Hz. Next, the values of synaptic stimulation were returned to control conditions and PSCs were recorded for 25 min (Noriega-Prieto et al., 2019) (Figure 4).

### Simulated spike

Simulated spike, after 5 min of stable baseline of EPSCs in voltage-clamp, after that, recording mode was switched to the current-clamp and a previously obtained neuronal spike was used as stimulus to depolarize the neurons through the recording pipette during 15 min at 0.2 Hz. Next, we were returned to control conditions and EPSCs were recorded for 25 min.

### Immunoelectron microscopy

After deep anesthesia with sodium pentobarbital (60 mg/kg), 6 month-old male mice (C57BL6/J) were perfused transcardially with 0.1 M phosphate-buffered saline (PBS), pH 7.4 followed by 4% paraformaldehyde, 75 mM lysine, 10 mM sodium metaperiodate in 0.1 M PB, pH 7.4. Brains were removed, post-fixed overnight in the same fixative solution at 4°C, coronally sectioned at 50 μm thicknesses on a vibratome (Leica VT1000S), and serially collected in wells containing cold PB and 0.02% sodium azide.

For IGF-IR immunogold labeling, sections containing the somatosensory cortex were used. Sections were first washed with PBS and incubated in a 50 mM glycine solution 5 min to increase antibody binding efficiency. Following a standard immunocytochemical protocol, tissue was first free-floating incubated in a rabbit polyclonal anti-IGF-IRα antibody (1/250, Santa Cruz) in a PBS 0.1 M/1% BSA solution for 48 h at 22°C. Then, sections were washed in PBS, and incubated with 1.4 nm gold-conjugated goat anti-rabbit IgG (1:100; Nanoprobes) overnight at 22°C. After post-fixing with 2% glutaraldehyde and washing with 50 mM sodium citrate, labeling was enhanced with the HQ Silver^™^ Kit (Nanoprobes), and gold toned. Finally, immunolabeled sections were fixed in 1% osmium tetroxide, block stained with uranyl acetate, dehydrated in acetone, and flat embedded in Araldite 502 (EMS, United States). Selected areas were cut in ultrathin sections (70–80 nm) and examined and photographed with a JEOL JEM1400 electron microscope. As a control for the immunogold technique, sections were processed as above but omitting the primary antibody. No specific labeling was observed in these control sections. We identified excitatory and inhibitory synapses based on the morphological appearance of the PSD in EM images. Excitatory (asymmetric) synapses typically display a pronounced post-synaptic density (PSD) much thicker than the relatively faint presynaptic thickening, while the PSD in inhibitory (symmetric) synapses looks similar to the presynaptic membrane (any synapse with a less marked PSD, similar to the presynaptic thickening, was classified as “symmetric”) (Gray, 1969).

### Immunohistochemistry

C57BL6/J mice (15 days old) were anaesthetized with pentobarbital (60 mg/kg) and perfused transcardially with 4% paraformaldehyde in 0.1 M phosphate buffer pH 7.4 (PB). The brain was then removed and post-fixed overnight. Coronal 50 μm-thick sections were cut and collected in PBS. Free-floating brain sections were pre-incubated in PB with Triton X-100 1% and bovine serum albumin 1%, and incubated overnight at 4°C with the respective primary antibody [mouse anti-PSD95 (1:500), mouse anti-vGAT (1:50) and rabbit anti-IGF-IR (1:100)]. Incubations were performed in two steps (first, PSD95/vGAT and then IGF1R). The following primary antibodies were utilized: a mouse anti-PSD95 antibody (Thermo Scientific 1:500), a mouse vGAT (Synaptic System 1:50) and a rabbit anti-IGF1R antibody (Santa Cruz, 1:100). Secondary Alexa-labeled antibodies from Molecular Probes (Donkey anti-mouse 488 Alexa-conjugated for the PSD95 and vGAT detection and donkey anti-mouse 594 Alexa-conjugated for the IGF1R detection) were used at a final concentration of 1:1000. All sections were counterstained with Hoechst (Calbiochem, 1:500). The incubation periods used were 1 h at room temperature (RT) and 48 h at 4°C for primary antibodies, 1 h RT and 24 h at 4°C for secondary antibodies, and 15 min for Hoechst incubation.

### Data analysis

Pre- or post- synaptic origin of the synaptic plasticity induced by IGF-I was tested by analyzing the modification in the variance that parallel the synaptic current amplitude change, which reflect the change in transmitter release probability (Clements, 1990; Malinow et al., 1990; Kuhnt and Voronin, 1994). To estimate the modification of the synaptic current variance, we first calculated the noise-free coefficient of variation (CV_NF_) of the synaptic responses before (control conditions) and during IGF-I. We used the formula CV_NF_ = √ (*δ*_synaptic current_^2^ − *δ*_noise_^2^)/*m*, where *δ*_noise_^2^ and *δ*_synaptic current_^2^ are the baseline and synaptic current peak variance, respectively, and *m* is the mean peak amplitude of the synaptic current. The ratio of the CV_NF_ (CV_R_) before (control conditions) and during IGF-I was obtained then for each neuron as CV_IGF-I_/CV_control_ (Clements, 1990). We constructed plots comparing variation in the normalized *m* (termed *M*) to the change in 1/CV_R_^2^ in each cell (Malinow et al., 1990). In these plots, depression of the synaptic currents has a presynaptic origin when values are below or in the diagonal, whereas points above the diagonal indicate a postsynaptic origin. However, potentiation of synaptic currents has a presynaptic origin when values are above or in the diagonal, whereas points below the diagonal indicate a postsynaptic origin (Faber and Kornt, 1991). This method requires a binomial EPSC amplitude distribution, a condition that must be met for the synaptic variance to reflect the probability of transmitter release. We could not directly test whether our data fitted a binomial distribution, but synaptic fluctuations were always evident and we assumed that synaptic release followed a binomial distribution. Data analysis was done in Clampfit 10 (Axon Instrument) and graphs were drawn in SigmaPlot 11. In all cases, statistical estimates were made with student’s two-tailed paired *t*-tests, and data are presented as means ± SE. To analyze the effects of different treatments and conditions, we carried out multiple comparison testing between the different groups using one-way ANOVA. The *post hoc* test used was Holm–Sidak or Dunn’s for parametric or non-parametric values respectively, versus control comparisons using the “after stimulus” situation and the “basal” condition as the controls to compare with a Holm–Sidak’s or Dunn’s multiple comparisons test. Statistical differences were established with *p* < 0.05 (∗), *p* < 0.01 (∗∗) and *p* < 0.001 (∗∗∗) for student’s *t*-test and *p* < 0.05 (#), *p* < 0.01 (##) and *p* < 0.001 (###) for the *post hoc* Holm–Sidak and Dunn’s test. The sample size is shown as *n* = *x*/*y* (*x* shows the number of cells, and *y* shows the number of animals).

## Results

### IGF-I regulates the induction of Hebbian LTP (LTP_H_) in a concentration dependent manner

We have previously shown that 10 nM IGF-I decreases the induction threshold of LTP_H_ (Noriega-Prieto et al., 2021). Here, we analyzed the effect of a slightly lower dose of IGF-I on LTP_H_ induced by spike timing dependent plasticity (STDP) at layers 2/3 of the barrel cortex (Figure 3A). We first checked in control conditions, the STDP protocol, consisting in a subthreshold PSP followed by a back-propagating action potential (BAP) at delays of 10 ms repeated 50 times at 0.2 Hz (Figure 3B) and then we performed similar experiments in slices in which previously IGF-I at doses of 10 nM, 7 nM or 5 nM was bath applied during synaptic stimulation of layer 4 at 0.2 Hz (Figure 3C). In control conditions, the STDP protocol induced an LTP_H_ of PSPs (from 96.01 ± 4.12 to 137.62 ± 8.33% of amplitude, *p* < 0.001; *n* = 7/3; Figures 3D,G, LTP control). However, in slices pretreated with 10 nM IGF-I, the STDP protocol induced a more robust LTP_H_ of PSPs (from 99.36 ± 1.08 to 141.59 ± 4.38% of amplitude after 20 pairings, *p* < 0.001; *n* = 6/2 and from 99.36 ± 0.89 to 169.13 ± 6.36% of amplitude after 50 pairings, *p* < 0.001; *n* = 6/3; Figures 3E,G, LTP facilitated). Conversely, in slices pretreated with 7 nM IGF-I, the STDP protocol did not induce LTP_H_ of the PSPs (from 101.66 ± 1.12 to 99.85 ± 6.35% of amplitude after 50 pairings, *p* > 0.05; *n* = 6/3; Figures 3F,G, LTP impairment). Interestingly, 7 nM IGF-I with 100 pairings repetition induced an LTD (from 102.74 ± 1.26 to 72.65 ± 5.19% of amplitude after 100 pairings, *p* < 0.001; *n* = 5/2; Figures 3F,G, LTP impairment), which further supports the idea of different IGF-I levels differently regulate the synaptic plasticity. Additionally, 50 pairings of pre- postsynaptic neuronal activity in the presence of picrotoxin (50 μM) and CGP (1 μM) to isolate the excitatory synaptic transmission, after 10 nM IGF-I did not induce any modulatory effect in the excitatory postsynaptic currents (EPSCs) (from 100. 57 ± 0.63 to 105.25 ± 10.13% of amplitude after 50 pairings; *p* > 0.05, *n* = 6/3; Supplementary Figures S2A–D, 50 pairings). Thus, only 10 nM, but not 7 nM IGF-I favored the induction of LTP_H_ through the modulation of inhibitory synaptic transmission (Noriega-Prieto et al., 2021). In other words, the activation of IGF-IRs favors or impairs LTP_H_ depending on IGF-I concentration.

### Bidirectional modulation of synaptic transmission by IGF-I levels

**Figure 5:**
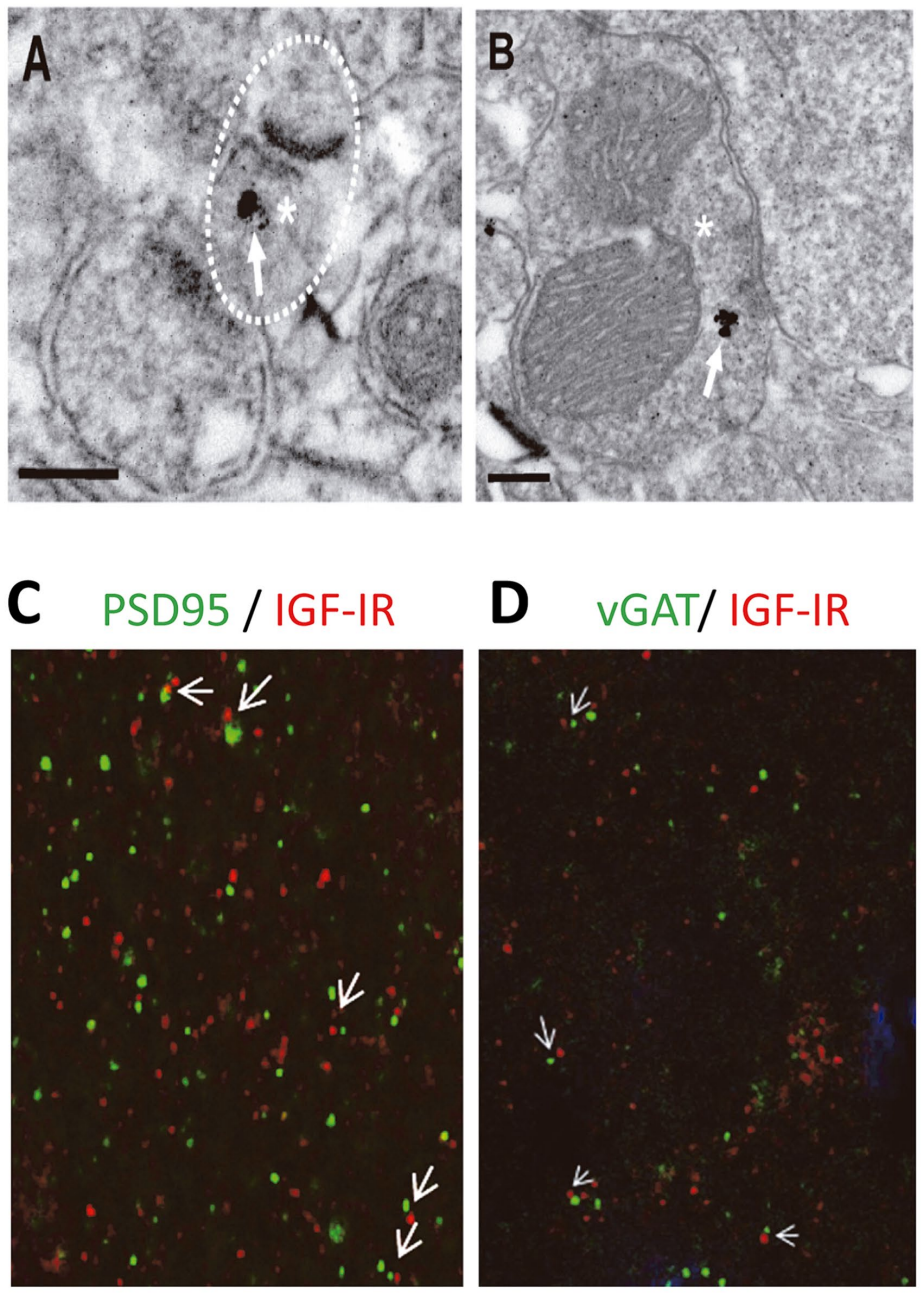
IGF-IRs are present at presynaptic sites of both excitatory and inhibitory synapses. Electron micrographs demonstrating IGF-IR silver enhanced immunogold labeling (white arrows) within both excitatory **(A)** and inhibitory **(B)** synapses. Immuno-particles were found associated to presynaptic terminals **(A,B)**. Asterisks indicate presynaptic terminals. Scale bars, 0.2 μm. Apposition of IGF-IR immunoreactive puncta (red) with both the postsynaptic marker PSD95 (green, white arrowheads in **C**) and the presynaptic marker vGAT (green, white arrowheads in **D**) agree with localization of IGF-IR at both sides of the synapse. DAPI staining of cell nuclei (blue).

We next compared the effects of these two doses of IGF-I on synaptic transmission. For this set of experiments, we recorded at L2/3 PNs (Figure 1A) the postsynaptic potentials (PSPs, Figure 1B) and currents (PSCs, Figure 1D top) evoked by stimulation of layer 4. After 5 min of recording PSPs and PSCs, 10 nM or 7 nM IGF-I was applied during 35 min. As previously published (Noriega-Prieto et al., 2021), 10 nM IGF-I induced a long-term potentiation (LTP) of the PSPs (termed LTP_IGF-I_) that remained 30 min after IGF-I washout (from 100.70 ± 1.58 to 127.50 ± 8.06% of amplitude at 60 min after IGF-I, *p* < 0.01, *n* = 8/3; Figures 1B,C, 10 nM IGF-I). However, the PSCs were transiently potentiated, returning to control values after IGF-I washout (from 98.58 ± 0.52 to 100. 29 ± 5.89% of amplitude 60 min after IGF-I, *p* > 0.05, *n* = 9/3; Figure 1D, IGF-I 10 nM, white circles). Interestingly, 7 nM IGF-I induced a long- term depression (LTD) of the PSPs (from 104.46 ± 0.82 to 70.10 ± 5.20% of amplitude 60 min after IGF-I, *p* < 0.01, *n* = 5/2; Figures 1B,C, 7 nM IGF-I) and the EPSCs (termed LTD_IGF-I_ from 103.78 ± 1.23 to 66. 22 ± 9.21% of amplitude 60 min after IGF-I, *p* < 0.05, *n* = 5/2; Figure 1D black circles and 7 nM IGF-I). Moreover, neither the PSPs (from 99.37 ± 0.67 to 102. 74 ± 2.31% of amplitude 60 min after IGF-I, *p* > 0.05, *n* = 5/2; Figures 1B,C, 5 nM IGF-I) nor PSCs (from 101.17 ± 0.88 to 91.95 ± 4.60% of amplitude 60 min after IGF-I, *p* > 0.05, *n* = 6/3; Figure 1D grey circles, and IGF-I 5 nM) were modified when 5 nM IGF-I was bath perfused. These results demonstrate that IGF-I can induce LTP or LTD of the PSPs at L2/3 PNs of the barrel cortex in a concentration dependent manner.

We next analyzed the locus of expression of IGF-I mediated LTD of the EPSCs. We first studied whether it was paralleled by a decrease in the probability of glutamate release. We pharmacologically isolated the EPSCs by blocking GABA_A_ inhibition with PiTX, and applied 7 nM IGF-I (Supplementary Figure S1). Under these conditions, IGF-I induced a similar LTD of the ESPCs than IGF-I 7 nM, that lasted at least 40 min of recording (from 99.59 ± 0.44 to 67.36 ± 3.87% of amplitude, *p* < 0.001, *n* = 6/2; Supplementary Figure S1A, black circles). This effect was inhibited with the IGF-IR antagonist NPV-AEW 554 (from 100.37 ± 0.37 to 94.87 ± 3.67% of amplitude, *p* > 0.05, *n* = 5/2; Supplementary Figure S1A, white circles). To investigate the pre or postsynaptic locus of expression of this LTD, we analyzed the effect of IGF-I on the pair pulse ratio (PPR) and on the EPCS variance by measuring the coefficient of variation (CV). The effect of IGF-I on both the PPR (from 0.98 ± 0.01 to 1.28 ± 0.10 before and after IGF-I respectively, *p* < 0.05, *n* = 6/2; Supplementary Figure S1B left) and the analysis of 1/CV^2^ plots (linear correlation *R*^2^ = 0.974, Supplementary Figure S1B right) revealed that LTD_IGF-I_ was due to a decrease in the probability of release of glutamate.

### Cytosolic calcium levels determine the sign of IGF-I mediated synaptic plasticity

Neurons secrete IGF-I by an activity-dependent pathway of exocytosis, and a mild depolarization is sufficient to induce IGF-I secretion in olfactory bulb neurons (Cao et al., 2011). Therefore, we next tested whether changes in cytosolic Ca^2+^ levels of postsynaptic PNs were involved in IGF-I potentiation of the EPSC. We carried out similar experiments as before, but in the presence of the Ca^2+^ chelator BAPTA (20 mM) in the recording pipette (Figure 2A). Under these conditions, 10 nM IGF-I induced an EPSCs depression (43.90 ± 4.04% of baseline, *p* < 0.001, *n* = 6/3, Figure 2B, 10 nM IGF-I, green circles) rather than an EPSCs potentiation observed in the absence of BAPTA. This EPSC depression was absent with 7 nM IGF-I (from 98.79 ± 1.37 to 99.19 ± 2.23% of amplitude, *p* > 0.05, *n* = 6/3; Figure 2B, 7 nM IGF-I, blue circles). Moreover, the plasticity was abolished in the presence of the IGF-I receptor antagonist NPV-AEW 554 (from 99.42 ± 1.79 to 104.08 ± 4.39% of amplitude, *p* > 0.05, *n* = 5/2; Figure 2B, 10 nM IGF-I + NVP, light red circles). These results indicate that IGF-I mediate EPSCs potentiation or depression depending on the cytosolic Ca^2+^ level, being all these forms of synaptic plasticity dependent on the activation of IGF-IRs.

Synaptotagmin 10 (Syt10) acts as the Ca^2+^-sensor that triggers IGF-I exocytosis in olfactory bulb neurons (Cao et al., 2011). Thus, we prevented exocytosis by using the light chain of the B type botulinum toxin (i.e., Botox 0.5 μM), which inhibits the SNARE protein-mediated membrane fusion of endosome complexes, and tested whether IGF-I effects depend on exocytosis. Surprisingly, 7 nM IGF-I did not modulate the EPSCs (data not shown), while IGF-I 10 nM depressed the EPSCs under BOTOX (42.26 ± 8.63% of baseline, *p* < 0.01, *n* = 6/3; Figure 2C, BOTOX + IGF-I 10 nM), suggesting that higher IGF-I concentrations are required for LTD_IGF1_ under Botox. Interestingly, increasing IGF-I to 20 nM was able to induce the potentiation of the EPSCs (227.80 ± 20.43% of baseline, *p* < 0.001, *n* = 7/3; Figure 2C, BOTOX + IGF-I 20 nM) indicating that higher IGF-I concentrations are required to LTP_IGF1_ under Botox probably because it blocked the activity-dependent release of IGF-I from the postsynaptic neuron.

### Synaptic stimulation and spiking activity can induce IGF-I mediated synaptic plasticity

Since cytosolic calcium levels and exocytosis are determinant in the induction of LTD and LTP by IGF-I, we next tested whether increases of cytosolic calcium induced by synaptic stimulation could be enough to induce IGF-I-mediated synaptic plasticity. After 5 min of recording the EPSCs, we increased the intensity of synaptic stimulation (SSI, synaptic stimulation increase) until a PSP followed by an action potential was recorded (Figures 4A,B). Next, we maintained evoked these suprathreshold responses for 15 min by SSI, and then we turned the stimulation intensity back to control values. This protocol of stimulation induced a LTD of the EPSCs (from 101.35 ± 2.43 to 54.54 ± 8.43% of amplitude, *p* < 0.01, *n* = 5/2; Figures 4A,B, black circles, SSI) that was prevented with NVP (from 101.09 ± 0.91 to 94.33 ± 4.50% of amplitude, *p* > 0.05, *n* = 6/3; Figures 4A,B, white circles, SSI + NVP), or under Botox (from 100.33 ± 1.01 to 100.60 ± 2.43% of amplitude, *p* > 0.05, *n* = 6/3; Figures 4A,B, red circles, SSI + Botox). These results suggest that suprathreshold responses induced by SSI are sufficient to induce an IGF-I-mediated LTD of the EPSCs dependent on endosome exocytosis. Finally, we checked whether simulation of these suprathreshold responses (Figures 4C,D, Simulated Spike) by injecting them through the patch recording electrode could induce a similar LTD of the EPSCs. As shown in Figures 4C,D, simulation of suprathreshold responses for 15 min was enough to induce a presynaptic LTD of the EPSCs (40.56 ± 3.58% of baseline, *p* < 0.01, *n* = 5/2; Figures 4C–E purple circles, simulat. spike), that is prevented with NVP (3.25 ± 3.38% of baseline, *p* > 0.05, *n* = 5/2; Figures 4C,D blue circles, simulat. spike + NVP). These results suggest that suprathreshold responses can induce increases in postsynaptic calcium levels that trigger the release of IGF-I and subsequent induction of LTD of the EPSCs.

### IGF-IR is present at presynaptic terminals of both excitatory and inhibitory synapses

We finally analyzed whether IGF-IRs were present at presynaptic terminals of excitatory in the postsynaptic L2/3 PNs. Electron micrographs demonstrated IGF-IR silver-enhanced immunogold labeling (Figures 5A,B, white arrows) at presynaptic terminals of both excitatory (Figure 5A) and inhibitory (Figure 5B) synapses. Moreover, Double immunocytochemical analysis for pre (vGAT)- and post- (PSD95) synaptic markers and IGF-IR indicate the presence of immunopositive puncta opposing each other in close proximity (Figures 5C,D), in agreement with the presence of IGF-IR in both pre- and post-synaptic terminals. The presence of IGF-IR in the presynaptic terminals of both glutamatergic and GABAergic synapses supports the presynaptic LTD of the EPSCs induced by 7 nM IGF-I (present results) and the presynaptic LTD of the IPSCs generated by 10 nM IGF-I (Noriega-Prieto et al., 2021).

## Discussion

### Role of IGF-I in the modulation of Hebbian synaptic plasticity

**Figure 6:**
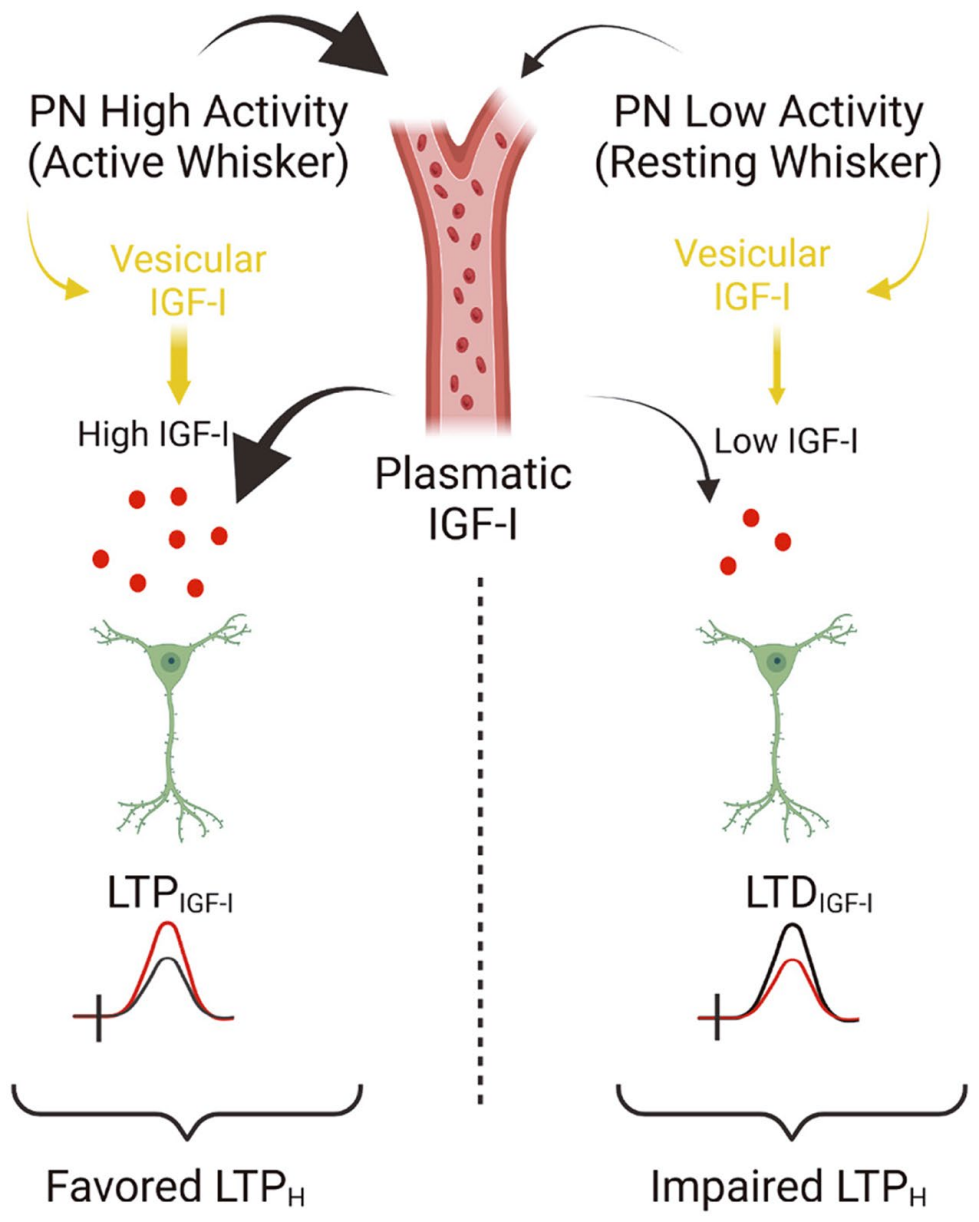
Model showing the concentration dependent actions of IGF-I. Simplified cartoon summarizing the novel results revealed in this work. **(Left part)** Under high extracellular levels of IGF-I (between 10 and 20 nM), occurring during high activity at pyramidal neurons (PN) of the barrel cortex during processing information of active whisker, LTP_IGF1_ is induced what favors Hebbian LTP and the associative learning and memory. Note that high extracellular IGF-I levels during spiking activity at barrel cortex are achieved by vesicular release of IGF-I stored inside PNs and maybe because facilitated IGF-I entry from plasmatic IGF-I. Indeed there is an increase in the uptake of IGF-I by the brain during whisker stimulation that correlates with frequency-dependent changes in cerebral blood flow in the barrel cortex (Nishijima et al., 2010). **(Right part)** On the contrary during low activity of barrel cortex PNs during resting whisker, both vesicular release of IGF-I and IGF-I entry from plasmatic levels should be lowered and then a lower IGF-I extracellular concentration is expected (below 10 nM) and LTD_IGF1_ is induced what impairs Hebbian LTP and the associative learning and memory (scheme was performed with BioRender).

In the central nervous system, IGF-I/IGF-IR signaling is critical for experience-dependent synaptic and neuronal plasticity in sensory cortices, adult neurogenesis (LLorens-Martín et al., 2010), synaptic vesicle release, and neuronal excitability (Nuñez et al., 2003; Maglio et al., 2021). Our present results challenge the standard view that IGF-I favors cognition by inducing LTP in cortical circuits, and it expands the range of brain IGF-I actions. Thus, we provide novel evidence that IGF-I levels determine the sign of plasticity induced at the barrel cortex; high levels induce LTP, while lower levels induce LTD, which in turn, favor or impair Hebbian LTP, respectively (Figure 6). While the reduction in the inhibitory tone by 10 nM IGF-I and the subsequent LTP of the synaptic plasticity reduces the threshold for inducing Hebbian LTP, the reduction of the excitatory transmission induced by 7 nM IGF1 would increase the threshold maybe by an insufficient activation of the necessary number of NMDARs to increase the cytosolic calcium to induce synaptic plasticity. Although the observation that IGF-I action on the brain is crucial for learning and memory is well established, here we are proposing an additional interpretation of the classical IGF-I mechanism. These results indicate that brain levels of IGF-I play an important role in synaptic plasticity, which is crucial for memory processes. Therefore, any changes in brain IGF-I levels could impact synaptic plasticity and cognitive functions.

### Depression of the excitatory synapses by IGF1

Extracellular IGF-I activates IGF-IRs under resting conditions, maintaining basal transmission and ongoing spiking activity in the physiological range, and induces synaptic modulation (Gazit et al., 2016). Presynaptic IGF-IRs are basally active, thus regulating glutamatergic synaptic transmission by modulating the glutamate release probability (Gazit et al., 2016). In fact, it can be concluded that tonic release of IGF-I and subsequent activation of IGF-IRs modulates synaptic vesicle release, leading to a short term depression in excitatory hippocampal neurons (Gazit et al., 2016). In agreement with these results, we also observed a long-term depression of synaptic transmission in the absence of postsynaptic activity (when firing at the recorded PN was prevented in voltage clamp) or when BAPTA abolished the cytosolic calcium increase mediated by this activity at PNs. Under Botox 7 nM IGF-I is not sufficient to induce LTD and 10 nM IGF-I is required for the LTD induction, whereas increasing the concentration to 20 nM IGF-I induces LTP. This demonstrates that endosome fusion is critical for establishing the concentration threshold for the induction of LTD and LTP by IGF-I, and suggests that exogenous application of IGF-I could induce neuronal-released IGF-I stored in endosomes (a calcium and endosome dependent IGF-I induce IGF-I release). These results strongly indicate that the tonic modulation of synaptic transmission induced by IGF-I described at the hippocampus is also present at glutamatergic synapses of the barrel cortex.

### IGF-I modulates both excitatory and inhibitory synaptic activity throughout the CNS

Our previous results show that 10 nM IGF-I induces an LTP of the PSPs, that we have termed LTP_IGFI_ (Noriega-Prieto et al., 2021). Moreover, we demonstrated that IGF-IR activation favors the induction of NMDAR-dependent LTP and improves texture discrimination of the mouse whiskers. Here we show that lower IGF-I levels (7 nM) induce LTD of the PSPs, that we have termed LTD_IGFI_ and impairs the induction of NMDAR-dependent LTP. These results are supported by the presence of IGF-IR in the presynaptic terminal of both excitatory and inhibitory synapses described in this work. In addition, based on the evidence, this mechanism of action has been studied in the hippocampus, where IGF-I can induce the release of GABA to regulate endogenous ACh release, possibly acting via GABAergic neurons (Seto et al., 2002). Moreover, olfactory learning in a social context selectively induces LTP of the GABAergic component of reciprocal synapses between granule and mitral cells in the medial olfactory bulb (MOB), requiring an autocrine and/or paracrine action of IGF-I to enhance postsynaptic GABA receptor function. Indeed, blocking Ca^2+^-triggered IGF-I release prevents GABAergic LTP (Liu et al., 2017). At any rate, our observations contribute to the notion that IGF-I modulates both excitatory and inhibitory synaptic activity throughout the CNS (Maglio et al., 2021; Noriega-Prieto et al., 2021). According to our results, IGF-I is able to induce a dual effect on glutamatergic synaptic transmission by the activation of IGF-IRs. In fact, both a pre-synaptically IGF-I-mediated potentiation and depression of the EPSCs were observed, and both were prevented by NPV-AEW 554 (Maglio et al., 2021; Noriega-Prieto et al., 2021).

### Bidirectional modulation of synaptic transmission by IGF-I

On the other hand, we also observed that IGF-I induced an increase in glutamatergic synaptic transmission through the activation of IGF-IRs. As discussed above, IGF-I-induced potentiation of excitatory synaptic transmission is dependent on the presence of exogenously applied IGF-I, thereby pointing to the importance of reaching specific local levels of IGF-I. Indeed, only IGF-I mediated depression of the EPSCs are observed by the release of IGF-I induced by high synaptic stimulation or simulated spikes (Figure 4). These results points to the importance of IGF-I uptake from the plasma in the induction of bidirectional plasticity, since only depression of the synaptic transmission can be induced without extracellular perfusion of IGF-I. Whereas there is a tonic IGF-I release that induces depression of glutamatergic synaptic transmission (Gazit et al., 2016), our results suggest that the bidirectional effect of IGF-I on the modulation of the EPSCs may depend on the levels of IGF-I reached (Figure 6). An IGF-I -mediated EPSC depression is produced when PNs fires during supra-threshold responses, whereas an IGF-I-mediated EPSC potentiation is induced by bath applied IGF-I. Under physiological conditions, higher levels of IGF-I could be reached in a neuronal-activity dependent manner (Nishijima et al., 2010). In fact, we described that serum IGF-I input to the brain is regulated by an activity-driven process that includes increased blood-brain barrier permeability to serum IGF-I (Nishijima et al., 2010). Also, it has been demonstrated that physical exercise induces increased brain uptake of serum IGF-I by specific groups of neurons throughout the brain (Carro et al., 2000; Nuñez et al., 2003). This increase in the uptake of IGF-I, under both mentioned physiological circumstances, induces an increase in neuronal excitability, which perfectly correlates with our previous *in vivo* results (Noriega-Prieto et al., 2021). The *ex vivo* investigation showed here suggests that this increase in IGF-I uptake would be essential in EPSC potentiation by increasing local IGF-I levels to those required for the modulation of synaptic transmission, playing a role in the increase in cortical activity.

In summary, IGF-I induces bimodal regulation of the excitatory and inhibitory synaptic transmission depending on its levels. This bidirectional action probably contributes to favor or impair the generation of associative memories impacting the barrel cortex-related behaviors. Although there is a SNARE dependent release of IGF-I from neurons (Cao et al., 2011) and Botox inhibits the SNARE protein-mediated membrane fusion of endosome complexes, the application of Botox, though effective in blocking the release of IGF-I stored in endosomes, can affect other neuromodulators stored in endosomes that modulate synaptic transmission, such as BDNF (Nasrallah et al., 2021), and interfere with glutamate receptor trafficking (Pellett et al., 2015). Additionally, further studies are necessary to investigate whether the bidirectional modulation induced by IGF-I is maintained in older animals. Despite potential limitations, our previous findings, together with the present work, reveal novel insights into the mechanisms of IGF-I signaling in the cortex.

## Significance statement

Insulin-like growth factor-I (IGF-I) signaling plays key regulatory roles in multiple processes of brain physiology, such as learning and memory, and brain pathology, such as Alzheimer disease. Yet, the underlying mechanisms remain largely undefined. Here we demonstrate that IGF-I signaling triggers long-term potentiation (LTP) or long-term depression (LTD) of synaptic transmission at cortical synapses in a concentration dependent manner, thus regulating the induction of Hebbian synaptic plasticity. The present work represents an important conceptual advance in our knowledge of the cellular basis of IGF-I signaling in brain function.

## Data availability statement

The original contributions presented in the study are included in the article/Supplementary material, further inquiries can be directed to the corresponding author.

## Ethics statement

The animal study was approved by the Ethical Committee of the Universidad Autónoma of Madrid, and Cajal Institute and are in accordance with Spanish (R.D. 1201/2005) and European Community Directives (86/609/EEC and 2003/65/EC). The study was conducted in accordance with the local legislation and institutional requirements.

## Author contributions

JN-P: Formal analysis, Investigation, Methodology, Supervision, Validation, Visualization, Writing – review & editing. LM: Data curation, Formal analysis, Methodology, Validation, Visualization, Writing – review & editing. JD: Data curation, Formal analysis, Validation, Writing – review & editing. AG: Data curation, Formal analysis, Validation, Writing – review & editing. IT-A: Conceptualization, Funding acquisition, Methodology, Validation, Writing – review & editing. DS: Conceptualization, Funding acquisition, Investigation, Methodology, Validation, Visualization, Writing – original draft, Writing – review & editing. PP-D: Writing – review & editing.

## Supplementary material

SUPPLEMENTARY FIGURE S1 LTD_IGF1_ is mediated by a long-term decrease in the glutamate release probability. **(A)** Time course of the EPSC recorded under PiTX before, during and after washing out IGF-I 7 nM in control (black circles, LTD_IGF1_) and under NVP (white circles). **(B left)** Summary data showing the paired-pulse ratio (R2/R1) of EPSCs recorded before (black bar, control) and during IGF1 7 nM (red bar, IGF-I). Note the increase in the paired-pulse ratio suggesting an increase in the glutamate probability of release. **(B right)** Plot of the variance (1/CV^2^, where CV is coefficient of variation) as a function of the mean peak EPSC amplitude in the presence of IGF-I normalized to control conditions (Mr). Note that the values were grouped following the diagonal suggesting that LTD_IGF1_ was due to a change in presynaptic release properties. ^*^*p* < 0.05, ^**^*p* < 0.01, and ^***^*p* < 0.001; student’s paired *t*-test.

SUPPLEMENTARY FIGURE S2 The spike-timing dependent protocol in 10 nM IGF-I does not elicit alterations in the synaptic transmission of EPSPs. **(A)** Representative responses recorded (PSP followed by an AP with a 10 ms delay and a frequency of 0.2 Hz) during the STDP protocol in control. **(B)** Time course scheme showing the IGF-I exposure, washout and STDP induction (black arrow). **(C)** Time course of the EPSP amplitude before and after STDP protocol in which 10 (green circles), 20 (purple circles) and 50 (orange circles) pairings were applied after 10 nM IGF-I. **(D)** Bar plot showing the relative EPSP peak amplitude baseline (pre-paring, grey bars) and 40 minutes after (post-pairing, light red bars) the application of the STDP protocol in which 10, 20 and 50 parings were applied after 10 nM IGF-I. ^*^*p* < 0.05, ^**^*p* < 0.01, and ^***^*p* < 0.001; student’s paired *t*-test. ^#^*p* < 0.01, ^##^*p* < 0.01, and ^###^*p* < 0.001; one-way ANOVA with post hoc Holm–Sidak; n.s., nonsignificant (*p* > 0.05).
