# Supplementary figures and images for: Bidirectional modulation of synaptic transmission by insulin-like growth factor-I
Source: Front Cell Neurosci. 2024 Jun 7;18:1390663. doi: 10.3389/fncel.2024.1390663 (PMC11193368; doi:10.3389/fncel.2024.1390663)

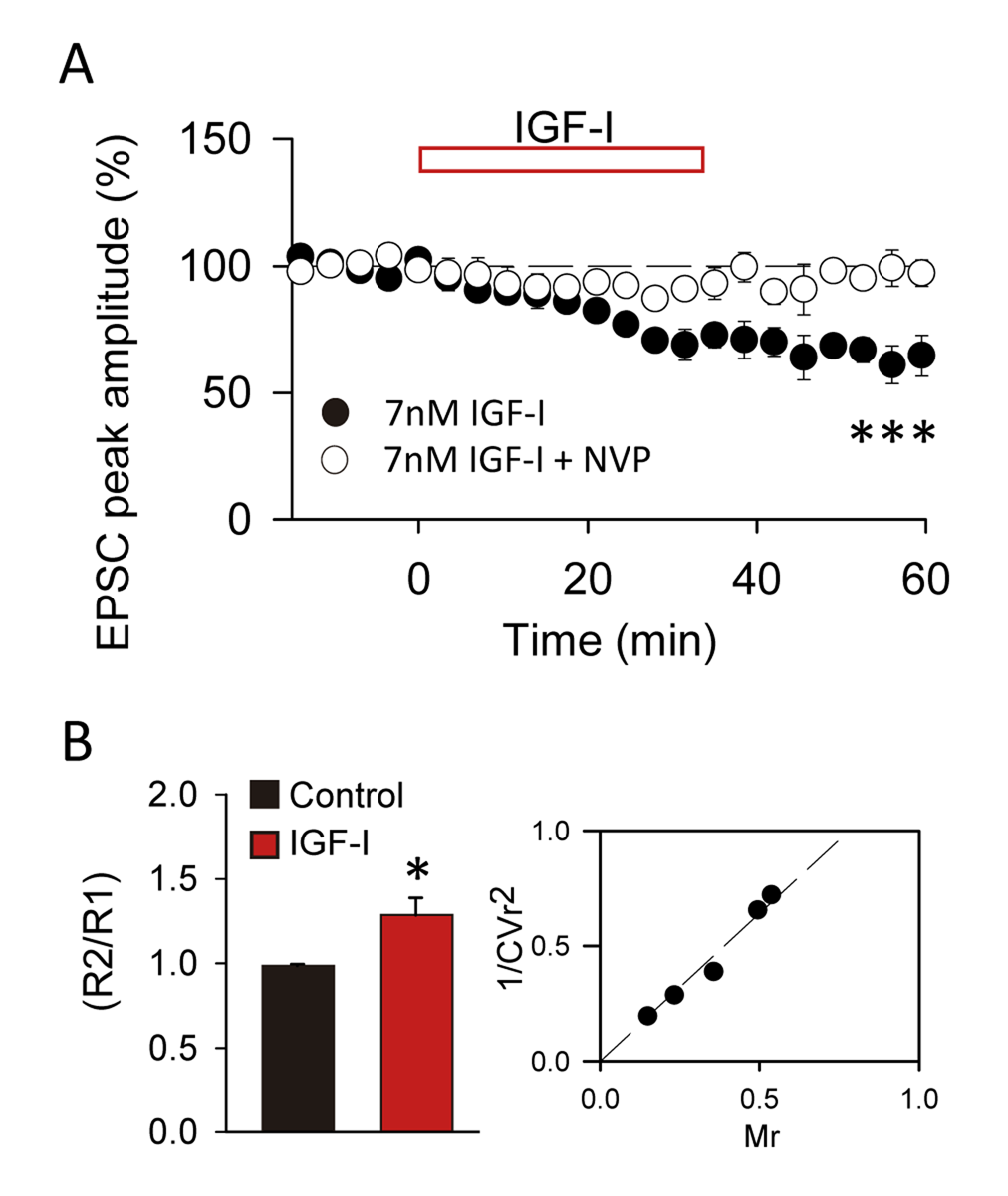

Supplement: SUPPLEMENTARY FIGURE S1 — LTDIGF1 is mediated by a long-term decrease in the glutamate release probability. (A) Time course of the EPSC recorded under PiTX before, during and after washing out IGF-I 7 nM in control (black circles, LTDIGF1) and under NVP (white circles). (B left) Summary data showing the paired-pulse ratio (R2/R1) of EPSCs recorded before (black bar, control) and during IGF1 7 nM (red bar, IGF-I). Note the increase in the paired-pulse ratio suggesting an increase in the glutamate probability of release. (B right) Plot of the variance (1/CV2, where CV is coefficient of variation) as a function of the mean peak EPSC amplitude in the presence of IGF-I normalized to control conditions (Mr). Note that the values were grouped following the diagonal suggesting that LTDIGF1 was due to a change in presynaptic release properties. *p < 0.05, **p < 0.01, and ***p < 0.001; student’s paired t-test. [file Image_1.tif]

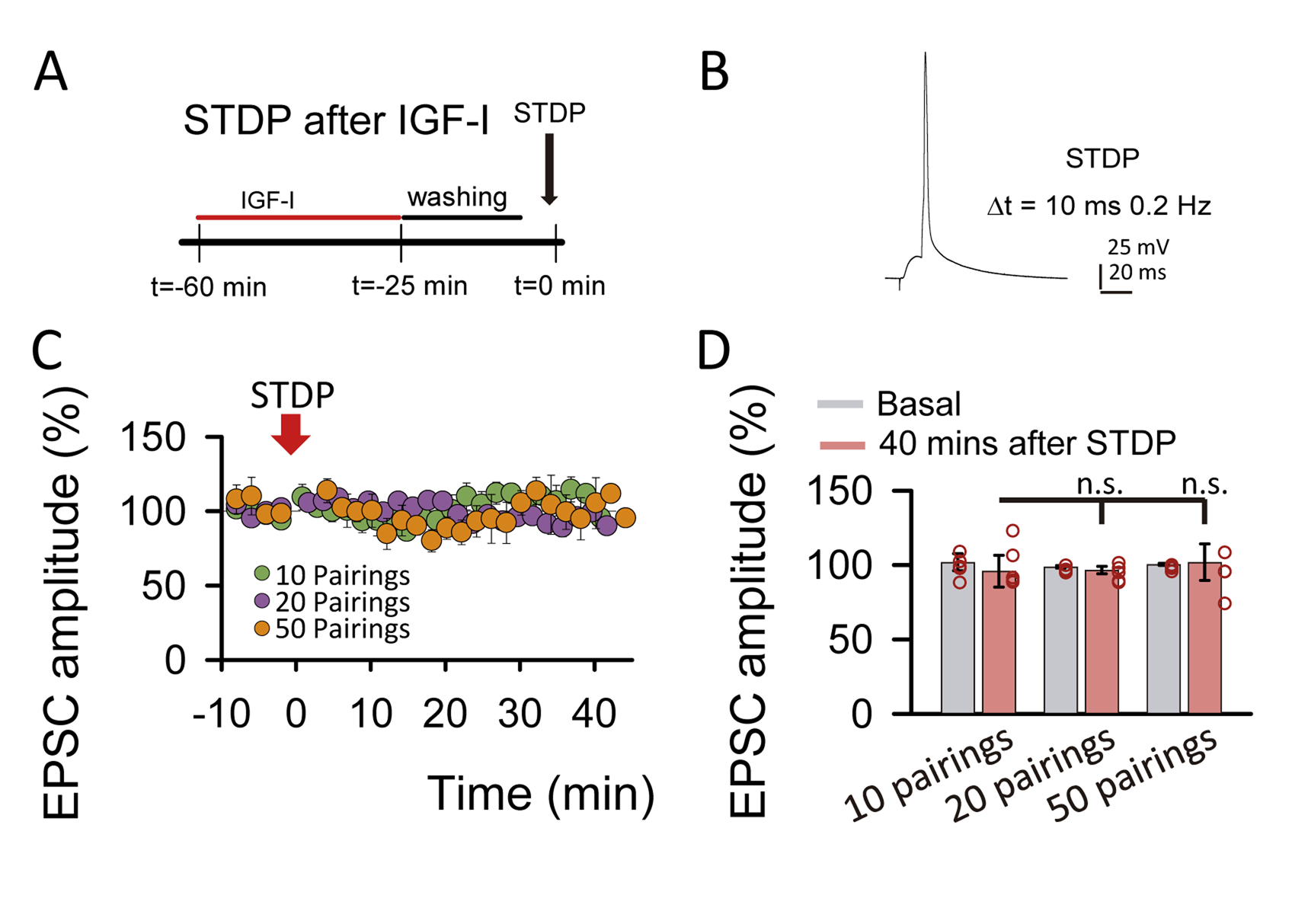

Supplement: SUPPLEMENTARY FIGURE S2 — The spike-timing dependent protocol in 10 nM IGF-I does not elicit alterations in the synaptic transmission of EPSPs. (A) Representative responses recorded (PSP followed by an AP with a 10 ms delay and a frequency of 0.2 Hz) during the STDP protocol in control. (B) Time course scheme showing the IGF-I exposure, washout and STDP induction (black arrow). (C) Time course of the EPSP amplitude before and after STDP protocol in which 10 (green circles), 20 (purple circles) and 50 (orange circles) pairings were applied after 10 nM IGF-I. (D) Bar plot showing the relative EPSP peak amplitude baseline (pre-paring, grey bars) and 40 minutes after (post-pairing, light red bars) the application of the STDP protocol in which 10, 20 and 50 parings were applied after 10 nM IGF-I. *p < 0.05, **p < 0.01, and ***p < 0.001; student’s paired t-test. #p < 0.01, ##p < 0.01, and ###p < 0.001; one-way ANOVA with post hoc Holm–Sidak; n.s., nonsignificant (p > 0.05). [file Image_2.tif]
